# Feeding fresh food and providing water ad libitum is clinically proven to exceed calculated daily water requirements and impact urine relative supersaturation in dogs

**DOI:** 10.3389/fvets.2025.1675990

**Published:** 2025-11-07

**Authors:** Rae Sires, Ryan Yamka, Joe Wakshlag

**Affiliations:** The Farmer’s Dog Inc., New York, NY, United States

**Keywords:** canine, fresh food, total water intake, water requirement, hydration, drinking

## Abstract

A series of studies were conducted to evaluate a fresh food for its impact on total water consumption compared to a dry or canned pet food and urine relative supersaturation in healthy adult dogs. *Study 1:* Ten dogs were used in a single cross-over study design to quantify and compare feeding a fresh food (71.1% moisture) versus a dry kibble food (6.1% moisture) on total daily water consumption (drinking + food moisture). Dogs consuming the fresh food consumed more food by weight on an as-fed basis (+348 grams; *p* < 0.0001), but less on a dry matter basis (−17 grams; *p* < 0.0001). Dogs fed the dry food consumed more water ad libitum (+276 grams; *p* < 0.0001) when compared to the fresh test food group, however dogs consuming the fresh food consumed significantly more total water daily on average per day (+88 grams; *p* < 0.0001). Dogs consuming the fresh food far exceeded their minimum water requirement compared to the dry food (141% vs. 102%; *p* < 0.0001). *Study 2:* Ten dogs were used in a single cross-over study design to quantify and compare feeding a fresh food (71.1% moisture) versus a retorted canned food (75.1% moisture) on total daily water consumption. The mean daily food consumption was statistically significantly higher for the dogs consuming the canned food compared to the fresh food (497 grams compared to 461 grams; *p* < 0.05). The total water intake of the canned food group was significantly higher than dogs fed the fresh control food (*p* < 0.05), however, all dogs had total water intakes well above 100% of their calculated water requirement. *Study 3:* Ten adult dogs were used to evaluate urine relative supersaturation; the mean for struvite was 0.203 ± 0.105 and the mean for calcium oxalate was 1.784 ± 2.660. In conclusion, fresh food can impact urine relative supersaturation and help support hydration in healthy adult dogs or those that are at risk of dehydration and water loss.

## 1 Introduction

Water is an essential nutrient and critical for normal physiologic functions in both healthy and diseased states in dogs, yet the ideal state of hydration or optimal hydration has not been fully defined (1). Dogs are triggered to drink when there is a central increase in plasma osmolality; therefore dogs generally operate in a state of mild dehydration and are reactive, rather than proactive, in drinking to maintain hydration (2).

Total daily water intake is the sum of three components: dietary water, voluntary consumption (drinking), and water produced during cellular metabolism and oxidation of macronutrients. Water requirements have been previously documented to be impacted by age and reproductive status (i.e., lactation), environmental factors, and disease states (1). Clinically, it is common to advise dog and cat owners to add water to their animal’s diet to increase water intake and promote diuresis, particularly in cases of crystalluria or urolithiasis (3). Feeding a high-moisture food (>75%) is recommended over feeding dry food (3), and one short-term study showed a diet with 73% moisture can increase total water intake relative to a dry kibble (7% moisture) in Miniature Schnauzers (4). However, these results contradict previous studies that demonstrated total water intake did not differ in dogs fed a dry or canned diet (5, 6) or dogs fed canned diets actually consume less total water due to a reduction in free-water intake (2, 7). There are also multiple studies that demonstrated either a reduction or no change in total water intake in cats consuming dry food compared to wet foods (5, 6, 8–10).

The pet food industry is continually evolving to meet the nutritional needs of dogs while simultaneously considering owner needs and desires for particular products. Pet food feeding trends commonly follow those of human foods, and there has been a recent push to consider the impacts of processing and highly processed foods on nutrition and overall health for both humans and animals. Owners are becoming more likely to seek out complete and balanced dog diets that include whole ingredients with less processing or artificial preservatives when compared to more traditionally manufactured canned or extruded products (11). The Association of American Feed Control Officials (AAFCO), an organization that proposes pet food definitions and guidelines, has not yet developed language to describe complete and balanced foods that are fully cooked, complete and balanced products but have been processed using less conventional methods. ‘Fresh food’ is commonly used in the industry to describe this group of products.

All previous protocols studying total water intake in dogs have evaluated either dry kibble diets with added water to adjust the dietary moisture, or used diets manufactured with variable water content (semi-moist or canned food formats), however, none have evaluated the impact of feeding a commercially available fresh food. While fresh, complete and balanced dog foods generally provide a similar moisture content to wet or canned products, it should not be assumed that these products perform similarly in a clinical setting. A series of studies was conducted to assess total water intake of a fresh food compared to an extruded, dry kibble diet or a retorted canned diet in healthy adult dogs. Additionally, a urinary study was conducted on the same fresh food to determine the impact on urinary relative supersaturation for struvite and calcium oxalate crystalluria.

## 2 Materials and methods

### 2.1 Care and use of animals

All research protocols were approved by a third-party Institutional Animal Care and Use Committee.

### 2.2 Water intake study 1

The purpose of this study was to determine the effect of a fresh food (The Farmer’s Dog Chicken & Grain Recipe) versus an extruded, dry kibble diet (Hill’s Science Diet Adult Chicken & Brown Rice No Corn, Wheat or Soy Dry Dog Food) on dietary moisture intake and total water consumption (free water intake plus dietary moisture intake) in adult dogs. The fresh food was prepared using human-grade ingredients and cooked to reach an internal temperature determined sufficient to act as a microbial kill step. The food was held at the predetermined temperature for a short period of time and then rapidly cooled to ensure food preservation during transport and storage prior to feeding.

Ten healthy adult beagle dogs were used in a single cross-over study design for a total of 14 days. The study consisted of two, 7-day phases. Dogs were grouped by sex, weight, and age prior to being equally divided into two groups (Group 1–5 dogs; Group 2–5 dogs). During Phase 1, Group 1 received the extruded, dry kibble diet (control diet), and Group 2 received the fresh food (test diet). During Phase 2, Group 1 received the fresh food, and Group 2 received the extruded, dry kibble diet. Dogs were fed once daily to meet their calculated maintenance energy requirement (MER) to maintain their current weight and body condition (MER = 1.8 × 70(BWkg)^0.75). All dogs were offered 1 liter (1,000 grams) of water at approximately the same time each day. If a dog consumed the entire 1 liter of water by the end of the workday, another 1 liter of water was offered. Water was weighed before and after being offered to ensure accuracy of tracking total intake. Daily food consumption and daily water consumption were recorded daily. All dogs were individually housed during feeding in a temperature-regulated facility with equal light–dark cycles (12 h each) according to USDA regulations.

Mean and standard deviation calculations were performed on daily food consumption, dietary dry matter intake, dietary water intake, free-choice water consumption, total water intake (free water intake plus dietary moisture intake), and calculated adequate fluid intake based on dry matter intake.

Other measured variables included moisture analysis of the control and test diets submitted to a third-party laboratory, weekly body weights, and daily observations including any potential adverse reactions.

The difference between diet groups was assessed for the average values by ANOVA modeling. The model included diet, sequence, period, day and diet-by-day interaction as fixed effects and dog within sequence as a random effect. LS mean values and between diet group *p*-value were obtained from the model. The residuals from the model were assessed for normality by the Shapiro–Wilk test and HoV by the Brown-Forsythe test. If either test was found to be statistically significant (*p* < 0.05) then a rank-transformation was applied prior to generating the ANOVA model.

Details for the diets used in this study are provided in Supplementary Table 1.

### 2.3 Water intake study 2

The purpose of this study was to determine the effect of a fresh food (The Farmer’s Dog Chicken & Grain Recipe) versus an retorted canned diet (Hill’s Science Diet Adult Chicken & Barley Entrée Dog Food Canned) on dietary moisture intake and total water consumption (free water intake plus dietary moisture intake) in adult dogs.

This study was conducted with the same study design and statistical analysis as mentioned above in section 2.1.

Details for the diets used in this study are provided in Supplementary Table 1.

### 2.4 Urinary relative supersaturation study

The purpose of the study was to assess the effects of a fresh food (The Farmer’s Dog Chicken & Grain Recipe) on urine pH and RSS when fed to healthy adult dogs.

Ten adult dogs were fed the fresh food for a period of 23 days. All dogs were pair housed except when being fed and during the 24 h urine collection period. Housing was temperature-regulated and had equal light–dark cycles (12 h each) according to USDA regulations.

Dogs were weighed weekly and feeding amounts were adjusted accordingly to maintain current body weight. Beginning on Day 22 of the study, dogs were separated into individual kennels to allow a 24-h urine sample to be collected from each dog for RSS analysis. On Day 23 of the study, total urine volume was measured, and urine pH and urine specific gravity (USG) analyses were performed on the representative 24-h urine sample from each dog. The urine samples were processed and sent out for urine mineral and analyte analysis at the University of Tennessee. Oxalate and citrate were analyzed utilizing the 930 Metrohm Ion Chromatograph with MagIC Net software. Sodium, potassium, chloride, calcium, magnesium, phosphorus, ammonia, and creatinine were analyzed using COBAS® c501 from Roche Diagnostics. RSS analysis for struvite and calcium oxalate was performed by utilizing EQUIL 93b (12).

Descriptive statistics using mean and standard deviation calculations were performed on urine volume, urine pH, urine specific gravity (USG), and RSS values. RSS for struvite and calcium oxalate were compared to published values for undersaturation, metastability, and oversaturation (<1, 1–14, and >14, respectively) (13, 14, 20).

Details for the diets used in this study are provided in Supplementary Table 1.

## 3 Results

### 3.1 Water intake study 1

A third-party analysis showed the control food, Hill’s Science Diet Adult Chicken & Brown Rice No Corn, Wheat, or Soy Dry Dog food, contained 6.1% moisture, and the test diet, The Farmer’s Dog Chicken and Grain, contained 71.1% moisture. Based on Shapiro-Wilks tests, the change and percent change in body weight over the testing period were determined to be normally distributed. The remaining data collected was not normally distributed. The mean average daily food consumption when dogs were offered the kibble diet, was 181 g (99% consumption) and was significantly lower (*p* < 0.0001) when compared to dogs who were offered the fresh food was 528 g (98% consumption). The mean change in body weight between the two diets was not statistically significant (−0.02% for the control diet compared to −0.47% for the test diet) and no significant adverse events were reported. Dogs consuming the fresh food had significantly higher average food consumption (*p* < 0.0001), water intake from food (*p* < 0.0001), total water intake (*p* < 0.0001), and significantly lower average ad libitum water intake (*p* < 0.0001) and lower average dry matter intake (*p* < 0.0001). Total fluid intake for the fresh food was well above 100% of the calculated fluid intake required based on dry matter intake with the fresh food being significantly higher (*p* < 0.0001). See Table 1 for the full data set.

**Table 1:** Study 1: food and water consumption for dogs fed a fresh food or an extruded, dry kibble diet.

Item	Fresh, human grade food		Extruded, dry kibble diet		Treatment (*p*-value)
	Mean	St. Dev.	Mean	St. Dev.	
Average food consumed, g	528.0	57.4	180.5	27.9	<0.0001
Average dry matter intake, g	152.6	16.6	169.5	26.2	<0.0001
Water intake from food, g	375.4	40.8	11.0	1.7	<0.0001
Average *Ad Libitum* water intake, g	147.1	85.6	423.4	81.0	<0.0001
Total Water Intake (Food and *Ad Libitum*), g	522.5	66.8	434.1	81.9	<0.0001
Calculated adequate fluid intake based on dry matter intake [dry matter intake (g) × 2.5 g], %	141	28.3	102	21.3	<0.0001

### 3.2 Water intake study 2

A third-party analysis showed the canned diet, Hill’s Science Diet Adult Chicken & Barley Entrée Dog Food Canned, contained 75.1% moisture, and the fresh food, The Farmer’s Dog Chicken and Grain, contained 71.1% moisture. All collected data was determined to be normally distributed based on Shapiro-Wilks tests. The mean average daily food consumption when dogs were offered the canned diet (497 g) was not significantly different when compared to the dogs that were offered the fresh food (461 g). However, when comparing the total food consumed to the total amount of food that was offered to each dog, dogs consuming the fresh food consumed 93% of what was offered compared to 79% when fed the canned food (*p* < 0.05). The mean weight change when dogs were offered the canned diet was −0.19 kg (−2.02%) and the mean weight change when dogs were offered the fresh food was −0.16 kg (−1.93%). The group mean average body weight change was not statistically significant when the test diet was compared to the control diet and no significant adverse events were reported.

No significant differences were found for average dry matter intake, water intake from food, average ad libitum water intake or total water intake. Total fluid intake of both diets well above 100% of the calculated fluid intake required based on dry matter intake with the retorted canned food being significantly higher (*p* < 0.05). See Table 2 for the full data set.

**Table 2:** Study 2: food and water consumption for dogs fed a fresh food or retorted canned diet.

Item	Fresh, human grade food		Canned retorted diet		Treatment (*p*-value)
	Mean	St. Dev.	Mean	St. Dev.	
Average food consumed, g	461	112	497	125	NS
Average dry matter intake, g	130	32	124	31	NS
Water intake from food, g	331	80	373	94	NS
Average *Ad Libitum* water intake, g	166	55	163	78	NS
Total Water Intake (Food & *Ad Libitum*), g	497	112	536	161	NS
Calculated adequate fluid intake based on dry matter intake [Dry matter intake (g) × 2.5 g], %	155	16	178	20	<0.05

NS, Not significant.

### 3.3 Relative supersaturation study

The 24-h mean urine pH was 6.53 ± 0.18, mean USG was 1.031 ± 0.009, and mean urine volume was 222 ± 128 mL. The mean RSS results were as follows: struvite 0.203 ± 0.105; calcium oxalate 1.784 ± 2.660. All dogs were determined to be within the undersaturated zone for struvite and on the lower end of the metastable zone for calcium oxalate.

## 4 Discussion

The dogs fed the fresh food consumed more food by weight on an as-fed basis compared to the dry kibble diet (528.0 grams compared to 180.5 grams, respectively), however they consumed less food by weight on a dry matter basis (152.6 grams compared to 169.5 grams). This is due to the significant difference in moisture content and macronutrient distribution between the two foods. The fresh food diet has a higher caloric density on a dry matter basis compared to the dry diet which allows dogs to consume less food by weight on a dry matter basis while maintaining the same total calorie intake.

Dogs fed the dry kibble food consumed more water ad libitum (i.e., drinking from their water bowl) when compared to the fresh food (423 grams vs. 147 grams; *p* < 0.0001), which can be explained by the drastic contrast in moisture content between the dry and fresh foods (6.1 to 71.1%, respectively), as dogs have been previously documented to adjust their free water consumption based on the moisture content of their food to achieve a relatively constant total water intake (5, 6). However, unlike previous studies in which dogs reduce their water intake to maintain a relatively constant intake of water, the dogs consuming the fresh food consumed significantly more water per day from dietary moisture plus free-choice water consumption compared to dogs eating the dry kibble diet (523 grams vs. 434 grams; *p* < 0.0001). A study by Stevenson et al. in 2003 demonstrated a difference in total water intake when Miniature schnauzers were fed a dry diet with 7% moisture or the same diet supplemented with deionized water to result in a 73% moisture content. These dogs consumed fewer calories compared to our study (110 kcal per kilogram of metabolic body weight), which would be expected to reduce total water intake directly due to reduced dietary intake. However, the dogs were also fed twice daily, compared to once daily in the current study, which may have contributed to increased total water intake as feeding triggers dogs to drink and have been documented to regularly drink 2–5 h after they have eaten regardless of the time they are fed and corresponding to meals (15, 16). The results of the higher moisture diet were in a different breed than what has been previously evaluated (Miniature schnauzers compared to Labrador retrievers) and the authors in that study concluded this may be due to breed variation.

In the study comparing total water intake between fresh food and canned food, both foods demonstrated total water intake was greatly increased relative to the calculated adequate water requirement per gram of dry matter. This outcome was expected for the fresh food due to the results of the first study demonstrating approximately 140% of total water intake compared to the calculated water requirement, however this was not expected for the canned food. Historically, dogs have demonstrated they compensate for the moisture content provided in their food and ultimately either maintain a relatively consistent total water intake or have a slight reduction in total water intake (2, 5–7). Both the canned food and the fresh food had similar moisture content (75.1 and 71.1%, respectively) and the canned food demonstrated a significantly higher total water intake compared to the fresh food. However, both foods resulted in total water intakes that were well above the calculated requirement when determined by dry matter intake.

The second study outcomes support the clinical recommendation to transition a dog to a food with a high moisture content to help support hydration for certain disease states. However, formulation of these diets to ensure that the patient is able to consume adequate volume of the product is essential as a high moisture content results in a reduced caloric density on an as-fed or metabolizable energy basis. All dogs through the study duration of the total water intake comparison of canned to fresh food were offered 100% of their calculated MER daily. Dogs fed the canned diet were offered a larger mean average daily weight of food compared to the fresh food group due to difference in dietary moisture and energy density. Dogs fed the canned food demonstrated an average consumption rate of 79% (ranging from 50 to 100%) of the food that was offered daily compared to 93% average consumption of the fresh food diet (ranging from 47 to 100%; *p* > 0.05). This difference and lower consumption in the canned food group may have been due to once-daily feeding and limitation to intake based on stomach volume. Over time, dogs may have become accustomed to the larger feeding volumes, or offering the diet in multiple meals may have been effective to increase total intake if stomach volume was the limiting factor in total intake. While no significant difference in weight changes were reported between the two groups, it is important to note that each food was only fed for a 1-week duration. If each phase of the study was continued longer than the current period, dogs fed the canned food and consuming 79% of their MER on a regular basis would be at a high risk of unintentional weight loss. The low consumption and need to consume a large portion of food to maintain weight and therefore lower percentage of intake relative to the food being offered may be desirable for select clinical indications, specifically obesity and a goal of weight loss, however dogs that are already at risk of dehydration due to other underlying disease processes may not be able to consume enough volume of the low energy density product in order to maintain weight. A food that has undergone AAFCO feeding trials, like the fresh food in this study, rather than being formulated to meet AAFCO guidelines, is preferable to ensure that the formulation and energy density is reasonable to ensure adequate energy and feeding intakes.

When calculating dogs’ total water intake and measuring the free-choice drinking volume in both total water comparative studies, evaporative losses and metabolic water production were not calculated in the total water intake from any of the three foods. Evaporative losses may have resulted in slight overestimation of water consumed from the dogs’ water bowls. However, these losses would have been similar between treatment groups and expected to minimally impact the comparison between products as the housing environment was controlled. Additionally, metabolic water was not considered in the animal’s daily water requirement. While metabolic water does contribute to the total water requirement of an animal, the animals in the current study were fed a consistent number of calories, which would have resulted in a similar production of metabolic water despite different macronutrient distributions (1). Estimated metabolic body water production was calculated separately from the study data and did not differ greatly between any of the treatment groups in either water intake study (Supplementary Table 2).

The addition of dietary sodium has been previously shown to increase water consumption in dogs and has a direct, linear correlation to the concentration of sodium in the diet (4–6, 17, 18). However, increased sodium may be contraindicated in some dogs dependent on health status (i.e., cardiac disease, calcium oxalate urolithiasis) and therefore may not be a viable nutritional strategy to increase water intake. The dry kibble, canned, and fresh food all had similar sodium concentrations (0.95 g, 0.83 g, and 0.86 g/1,000 kcal, respectively per information provided on the company website) and is not expected to be a driver to the overall higher total water intake by the dogs consuming the fresh or canned foods. While the addition of water supplements have been documented as being effective in increasing water intake and subsequent urine production (19), this method of management would require careful application by the owner as depending on the ingredients may predispose the water to additional contamination or bacterial growth. Some clinicians simply recommend adding tap water to the commercial diet, including canned products, to achieve a dietary moisture of 85% (20). However, the large volume of water that would be required to reach this level of moisture regardless of the starting diet format may negatively impact palatability, diet texture, and possibly result in gut fill due to the large amount of extra volume that was not anticipated or appropriately compensated for in formulation by the diet manufacturer.

In a final feeding trial, the fresh food was evaluated for impact on RSS for the risk potential for development of struvite and calcium oxalate crystal precipitation due to the consistently documented increased water intake in the previous two studies. The results in this study demonstrated that the RSS values for both struvite and calcium oxalate were consistent with either an undersaturated or metastable urinary environment, which can help aid in prevention and/or dissolution of crystals dependent on the crystal type present (13). While these parameters were not collected concurrently while measuring total water intake, the results suggest that the total water intake from the fresh food in both comparative studies is sufficient to positively impact urine RSS and may be beneficial for dogs with lower urinary tract disease or at risk of certain types of crystalluria. RSS evaluation was not performed on the control products of the previous studies due to resource limitations and those products not exhibiting marketing claims for urinary health.

There were a few limitations to these studies. The RSS and water consumption studies were not run concurrently or in the same subset of individual dogs, which prevents direct assessment of the increased total water intake impacting RSS values. The dogs in the hydration study were housed in a controlled environment and had access to free-choice water at all times. For this reason, the results of the hydration study may be appropriate to apply to pet dogs but less applicable to working dogs due to their varied work environments and potentially intermittent access to water. Finally, the dogs used in these studies were of a single breed, which may not account for any breed or size differences in the measured parameters or drinking behaviors.

In conclusion, a highly palatable fresh food can impact urine relative supersaturation and help support hydration in adult dogs by increasing total water intake relative to their calculated water requirements. If a high-moisture food is recommended to help manage a clinical condition, a food that has undergone AAFCO feeding trials is recommended to ensure that nutrient and energy needs can be adequately met. This series of studies supports that fresh foods offer an alternative food format to more traditionally processed foods while offering similar clinical benefits. The increase in total water consumption from consuming a fresh food may help support certain disease states that predispose an animal to increased water loss, those that benefit from increased water intake such as urolithiasis, or proactively support normal hydration in healthy and working dogs.

## Data Availability

The original contributions presented in the study are included in the article/Supplementary material, further inquiries can be directed to the corresponding author/s.
