# Supplementary material for: Feeding fresh food and providing water ad libitum is clinically proven to exceed calculated daily water requirements and impact urine relative supersaturation in dogs
Source: Front Vet Sci. 2025 Nov 7;12:1675990. doi: 10.3389/fvets.2025.1675990 (PMC12636039; doi:10.3389/fvets.2025.1675990)
Supplement: Supplementary file 1 [file Table_1.docx]

SUPPLEMENTAL MATERIAL:

Table 1: Nutritional Content of Diets from Water Intake Studies

|  | Diet A | Diet B | Diet C |
| --- | --- | --- | --- |
| % Fat, minimum | 4.5 | 19.0 | 3.0 |
| % Protein, minimum | 7.0 | 12.5 | 5.2 |
| % Fiber, maximum | 1.5 | 3.0 | 1.5 |
| % Moisture, maximum | 74.5 | 10.0 | 78.0 |
| Calcium (g/Mcal) | 4.24 | 2.18 | 2.03 |
| Phosphorus (g/(Mcal) | 2.98 | 1.91 | 1.67 |
| Sodium (g/Mcal) | 0.86 | 0.95 | 0.83 |

Diet A - The Farmer’s Dog Chicken and Grain Recipe; Diet B - Hill’s Science Diet Adult Chicken & Brown Rice No Corn, Wheat, or Soy Dry Dog food; Diet C - Hill’s Science Diet Adult Chicken & Barley Entrée Dog Food Canned
