# Supplementary material for: Feeding fresh food and providing water ad libitum is clinically proven to exceed calculated daily water requirements and impact urine relative supersaturation in dogs
Source: Front Vet Sci. 2025 Nov 7;12:1675990. doi: 10.3389/fvets.2025.1675990 (PMC12636039; doi:10.3389/fvets.2025.1675990)
Supplement: Supplementary file 2 [file Table_2.docx]

SUPPLEMENTAL MATERIAL:

Table 2: Estimated metabolic water production from the water intake study diets:

|  | Diet A* | Diet B | Diet C |
| --- | --- | --- | --- |
| Protein (g/1000 kcal) | 73 | 59 | 65 |
| Fat (g/1000 kcal) | 54 | 41 | 41 |
| Carbohydrate (g/1000 kcal) | 81 | 129 | 120 |
| Metabolic water** from protein (mL) | 30 | 24 | 27 |
| Metabolic water from fat (mL) | 58 | 44 | 44 |
| Metabolic water from carbohydrate (mL) | 49 | 77 | 72 |
| Total estimated metabolic water produced from 1000 kcal of diet (mL) | 137 | 145 | 143 |

* Diet A - The Farmer’s Dog Chicken and Grain; Diet B - Hill’s Science Diet Adult Chicken & Brown Rice No Corn, Wheat, or Soy Dry Dog food; Diet C - Hill’s Science Diet Adult Chicken & Barley Entrée Dog Food Canned

**Estimated metabolic water production was calculated using the factors of 41 grams, 107 grams, and 60 grams of water produced per 100 grams of protein, fat, and carbohydrate, respectively (1).

1. Rowntree LG. The water balance of the body. *Physiol Rev*. (1922) 2:116–69. doi: 10.1152/physrev.1922.2.1.116
